# Atypical E2fs Control Lymphangiogenesis through Transcriptional Regulation of Ccbe1 and Flt4

**DOI:** 10.1371/journal.pone.0073693

**Published:** 2013-09-12

**Authors:** Bart G. M. W. Weijts, Andreas van Impel, Stefan Schulte-Merker, Alain de Bruin

**Affiliations:** 1 Department of Pathobiology, Faculty of Veterinary Medicine, Utrecht University, Utrecht, The Netherlands; 2 Hubrecht Institute-KNAW and UMC Utrecht, Utrecht, The Netherlands; 3 EZO Department, University of Wageningen, Wageningen, The Netherlands; Feinberg Cardiovascular Research Institute, Northwestern University, United States of America

## Abstract

Lymphatic vessels are derived from venous endothelial cells and their formation is governed by the Vascular endothelial growth factor C (VegfC)/Vegf receptor 3 (Vegfr3; Flt4) signaling pathway. Recent studies show that Collagen and Calcium Binding EGF domains 1 protein (Ccbe1) enhances VegfC-dependent lymphangiogenesis. Both Ccbe1 and Flt4 have been shown to be indispensable for lymphangiogenesis. However, how these essential players are transcriptionally regulated remains poorly understood. In the case of angiogenesis, atypical E2fs (E2f7 and E2f8) however have been recently shown to function as transcriptional activators for VegfA. Using a genome-wide approach we here identified both CCBE1 and FLT4 as direct targets of atypical E2Fs. E2F7/8 directly bind and stimulate the *CCBE1* promoter, while recruitment of E2F7/8 inhibits the *FLT4* promoter. Importantly, inactivation of *e2f7/8* in zebrafish impaired venous sprouting and lymphangiogenesis with reduced *ccbe1* expression and increased *flt4* expression. Remarkably, over-expression of *e2f7/8* rescued Ccbe1- and Flt4-dependent lymphangiogenesis phenotypes. Together these results identified E2f7/8 as novel *in vivo* transcriptional regulators of *Ccbe1* and *Flt4,* both essential genes for venous sprouting and lymphangiogenesis.

## Introduction

The lymphatic vascular system is a specialized capillary network of blind ended vessels that are essential for maintaining interstitial fluid balance, macro-molecular uptake and immune cell trafficking. One of the main drivers behind lymphangiogenesis is the Vascular endothelial growth factor C (VegfC) – Vegf Receptor 3 (Vegfr3; Flt4) pathway [1]–[4]. Tight regulation of VegfC-Flt4 signaling is of fundamental importance for proper lymphangiogenesis. It has been shown that Delta like ligand 4 (Dll4) suppresses VegfC-Flt4 signaling while Collagen- and Calcium-binding EGF domains 1 (Ccbe1) enhances the biological effect of VegfC, thereby regulating the lymphangiogenic response in opposing ways [2], [5]. Besides these important findings, it currently remains unclear how these factors are regulated at the transcriptional level.

The atypical E2fs, E2f7 and E2f8, form homo- or heterodimers, possess two DNA binding domains and form thereby an unusual duo within the E2F family [6]–[8]. E2f7/8 function predominantly as transcriptional repressors of cell cycle genes involved in DNA replication, DNA metabolism, DNA repair, mitosis and cytokinesis [9], [10]. However, we recently showed that E2f7/8 can also function as a transcriptional activator of VegfA, thereby promoting blood vessel formation [11]. The aim of this study was to determine whether E2f7/8 modulate lymphangiogenesis through transcriptional regulation of lymphangiogenic factors. We report here that Flt4 and Ccbe1 are directly regulated by E2f7/8 and thereby show that these atypical E2Fs are essential modulators of lymphangiogenesis *in vivo*.

## Results

### E2F7/8 Directly Regulate CCBE1 and FLT4 Expression

**Figure 1:**
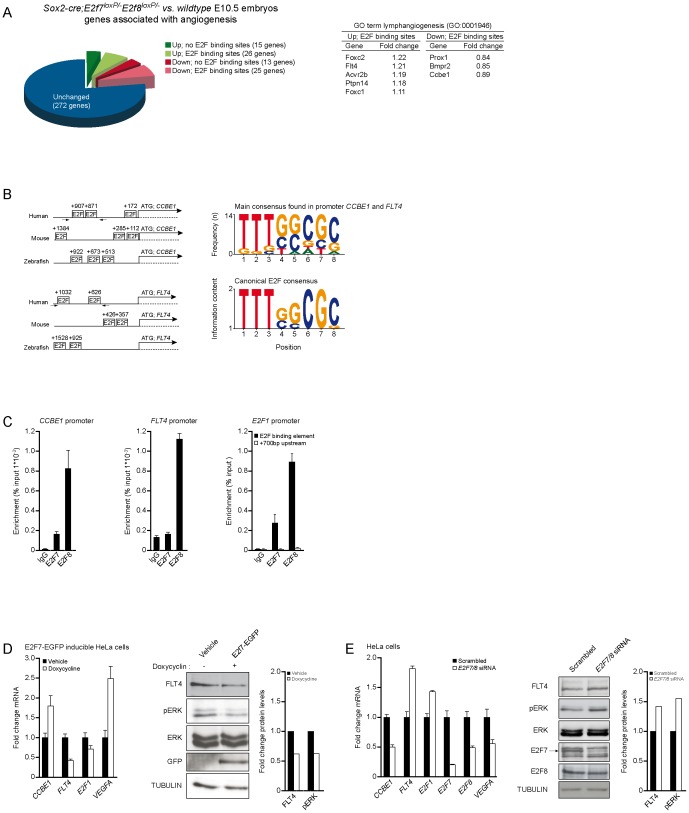
E2F7/8 directly regulate CCBE1 and FLT4 expression. A, Genes associated with the gene ontology (GO) term angiogenesis were extracted from the *Sox2-cre;E2f7^loxP/−^E2f8^loxP/−^* vs. *wild-type* E10.5 mouse embryos (*P*<0.05) database (GEO: GSE30488) and additionally analyzed for E2F binding sites and their presence in GO lymphangiogenesis (GO:0001946). B, E2F binding elements within the *CCBE1* and *FLT4* promoter. Average and canonical E2F binding consensus. C, E2F7 and E2F8 ChIP performed on *CCBE1* and *FLT4* promoters in HeLa cells, arrows in (B) indicate primers used for ChIP. A previous reported E2F site within the *E2F1* promoter and a non-specific site upstream (+700 bp) served as controls. D, mRNA and protein levels of E2f7-EGFP inducible HeLa cells treated with doxycyclin (0.2 µg /ml) for 24 hours. E, mRNA and protein levels of HeLa cells treated with scrambled or *E2f7/8* siRNAs. Fold change in protein were calculated using TUBULIN as reference. Data presented as the average (±s.e.m.) compared to the control condition in three independent experiments.

Recently, we showed that E2f7/8 regulate VegfA dependent angiogenesis in zebrafish [11]. To determine whether E2f7/8 also control other angiogenenic or lymphangiogenic factors besides VegfA, we searched in a recently published genome-wide microarray analysis on *E2f7/8*-deficient E10.5 mouse fetuses (*Sox2-cre;E2f7^loxP/−^;E2f8^loxP/−^*) [12] for de-regulated expression of genes associated with the AmiGO gene ontology (GO) term (lymph)angiogenesis (Figure 1A). This analysis revealed that among the genes that have been shown to be indispensable for lymphangiogenesis, only *Ccbe1* and *Flt4* were deregulated and contained canonical *E2f* binding sequences within their proximal promoter (Figure 1A, B) [2], [13]. To investigate whether these genes are indeed bound and regulated by E2F7/8, we first performed chromatin immunoprecipitation (ChIP) experiments in HeLa cells and found that both E2F7 and E2F8 bound strongly to the *CCBE1* promoter (Figure 1C). E2F8 was also strongly enriched on the *FLT4* promoter, while E2F7 showed only weak binding (Figure 1C), which might be due to the overall lower affinity of the E2F7 antibody. We used a previously reported *E2F* binding site within the *E2F1* promoter and a non-specific site upstream as controls (Figure 1C) [11]. Next we tested whether ectopic expression of E2F7 was able to modulate the expression of *CCBE1* and *FLT4*. To this extent we used HeLa cells which express E2F7-eGFP upon administration of doxycycline [10]. Induction of E2F7 showed an increase in *CCBE1* mRNA and a decrease in FLT4 mRNA and protein levels (Figure 1D). Additionally, phosphorylation of extracellular-signal-regulated kinase (pERK), a downstream factor of FLT4 signaling, showed a decrease while total ERK levels were unchanged (Figure 1D). As controls, two previously described atypical E2F target genes, E2F1 and VEGFA, were used (Figure 1D) [11]. Conformingly, knockdown (KD) of E2F7 or E2F8 as well as the combination of E2F7/8 caused a decrease in *CCBE1* mRNA levels, while *FLT4* mRNA and protein levels were increased (Figure 1E, Figure S1A). Consistently, downstream phosphorylation or ERK was increased upon deletion of *E2F7/8* (Figure 1E). The deregulation of CCBE1 and FLT4 was stronger by E2F7 KD and E2F7/8 KD compared to E2F8 KD, whereas E2F1 KD had no obvious effects (Figure S1A). Consistent with previous reports, E2F7 KD resulted in derepression of E2F8 expression, and E2F8 KD lead to derepression of E2F7 expression, indicating that atypical E2Fs can compensate for each other [11] (Validation of the siRNA is shown in Figure S1A).

Next, we investigated whether E2F7/8 regulate *CCBE1* and *FLT4* in cell types that reflect their *in vivo* expression pattern. As *ccbe1* is strongly expressed in mesenchymal cells in zebrafish (30 hpf) [13], we used mouse embryonic fibroblasts (MEFs). *Flt4* expression in venous endothelial cells is essential to mediate the budding of lymphatic precursors from the venous system [2]. In addition, *flt4* expression in lympathic endothelial cells (LECs) has been shown to be important for LEC proliferation upon stimulation with VEGFC *in vitro* [14]. Therefore, we used human umbilical vein endothelial cord cells (HUVECs) and human lymphatic endothelial cells (LECs) to investigate *FLT4* expression. Moreover, previous analysis on the spatio-temporal expression at the onset of lymphangiogenesis, revealed a ubiquitous pattern for atypical E2fs in mice (E9.5) and zebrafish (30 hours post fertilization (hpf)) [6], [11]. In line with these *in vivo* expression patterns, we found in comparison to HeLa cells, a 4000 times higher expression of *Ccbe1* in MEFs and a 230 and 890 times higher expression of *FLT4* in HUVECs and LECs respectively. In line with the reported expression of *CCBE1* [5], CCBE1 mRNA levels were nearly undetectable in LECs. In contrast, atypical E2Fs displayed a comparable expression level in all three cell lines (Figure S1B). Furthermore, we analyzed public available CHIP-sequencing data (ENCODE; unavailable for LECs) for the trimethylated Lys4 and Lys27 mark on histone H3, in which Lys4 methylation positively regulates transcription while Lys27 methylation negatively regulates transcription [15], [16]. Comparing the levels of H3K4me3 and H3K27me3 between HUVECs, HeLa cells and fibroblasts confirmed that the active transcription status of *E2F7, E2F8, CCBE1 and FLT4* promoters correlates with the cell-type specific expression for CCBE1 in mesenchymal cells and FLT4 in endothelial cells (Figure S1A). To test whether E2F7/8 are also capable of modulating CCBE1 and FLT4 expression in MEFs, HUVECs and LECs, we inactivated E2F7/8 in these cell lines utilizing a conditional deletion approach in *E2f7^loxP/loxP^ E2f8^loxP/loxP^* MEFs [6] and siRNA technology in endothelial cells. As expected, knockdown of atypical E2fs in MEFs and HUVECs resulted in decreased *CCBE1* expression, while *FLT4* showed an increased expression in all three cell types (Figure S1B).

Together, our results show that E2F7/8 directly bind and modulate *CCBE1* and *FLT4* expression in cell types that reflect their *in vivo* expression pattern. The *in vitro* expression and H3K4me3 and H3K27me3 CHIP-sequence data indicated that E2F7/8 are ubiquitous expressed between all cell lines used, while *CCBE1* and *FLT4* show a cell type specific expression pattern, mesenchymal cells and endothelial cells respectively. Therefore, we hypothesize that E2F7/8 do not provide an ON/OFF switch for tissue specific expression pattern of *CCBE1* and *FLT4*, but rather fine-tune the expression levels of these lymphangiogenesis factors.

### Loss of *e2f7/8* Impaired Venous Sprouting and Lymphangiogenesis

**Figure 2:**
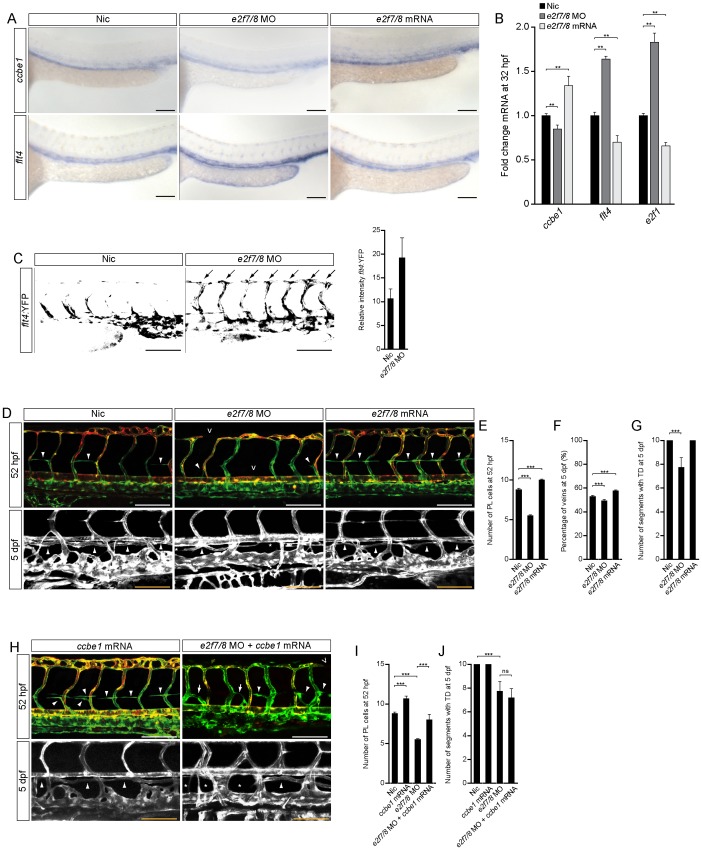
Loss of E2f7/8 impaired venous sprouting and lymphangiogenesis. A, *In situ* hybridisation and B, qPCR (** *P*<0.05; two independent experiments with n = 10 per condition and experiment) for *flt4* and *ccbe1* in zebrafish embryos 32 hpf, un-injected control (nic) or injected with *e2f7/8* MOs or mRNA. C, *Flt4*:YFP transgene level of 36 hpf uninjected or *e2f7/8* MOs injected embryos, lateral view (n = 30 per condition). D–G Lateral images and quantification of *Tg(fli1a:gfp;flt1^enh^:rfp*) embryos treated as indicated and imaged at 52 hpf or 5 dpf. H–J Lateral images and quantification of *Tg(fli1a:gfp;flt1^enh^:rfp*) embryos treated as indicated and imaged at 52 hpf or 5 dpf. Concentrations: *e2f7/8* MOs (10 ng each); *e2f7/8* mRNA (100 pg each); *ccbe1* mRNA (100 pg). Open arrow heads indicate missing intersegmental vessels or dorsal longitudinal anastomotic vessel. Closed arrow heads indicate PLs (upper panel) or the TD (lower panel). Arrows depict PLs that have connected to ISVs. Stars indicate missing TD fragments. All scale bars are 100 µm. Data presented as the average (±s.e.m.) compared to the control condition in three independent experiments (*** *P*<0.001). At least n = 150 embryos per condition in three independent experiments were used for D–J.

To investigate E2f7/8 function in lymphangiogenesis *in vivo*, we injected zebrafish embryos at the one-cell stage with full length *e2f7/8* mRNA or used morpholino oligomers (MOs) to prevent correct splicing of *e2f7/8*. As described previously, sequencing of MO induced miss-spliced *e2f7* and *e2f8* mRNA revealed the presence of frameshifts or intron insertion upstream of DNA binding domain [11]. Consistent with our *in vitro* studies, knockdown (KD) of E2F7/8 resulted in decreased *ccbe1* and increased *flt4* levels, while forced expression of *e2f7/8* mRNA had the opposite effect (Figure 2A, B). Furthermore, KD of *e2f7/8* in a transgenic line that drives YFP expression under the control of the *flt4* promoter showed 2-fold increased YFP expression, mainly apparent in the ISVs (Figure 2C).

In zebrafish, lymphangiogenesis starts around 36 hpf [13], [17] with cells that sprout from the posterior cardinal vein (PCV). A subset of these cells connects to the arterial intersegmental vessels (aISVs) and remodels them to venous ISVs (vISVs), which consequently determines the ratio of arteries and veins within the trunk vasculature. The “non-connecting” cells migrate further dorsally and populate the horizontal myoseptum (HM) and are referred to as parachordal lymphangioblasts (PLs) [13]. Next, these PLs start to migrate ventrally and dorsally from the HM exclusively alongside aISVs [17] and give rise to the embryonic lymphatic structures, including the thoracic duct (TD) [13].

We assessed E2f7/8 function during lymphangiogenesis by using *Tg(fli1a:gfp;flt1^enh^:rfp*) zebrafish embryos, in which endothelial cells (ECs) are labeled green and arterial ECs have an additional red color [17]. At 52 hpf, KD of *e2f7/8* resulted in a decreased number of PLs at the HM (Figure 2D upper panel; 2E). As described earlier, *E2f7/8* morphants also display defects or missing aISVs (Figure 2D upper panel) [11].

At 5 days post fertilization (dpf) we found a decreased number of vISVs in *e2f7/8* morphants (Figure 2F). Together with the reduced number of PLs at the HM, these findings indicates that the total number of cells initially sprouting from the PCV was reduced in *e2f7/8* morphants. Furthermore, the formation of the TD was impaired in *e2f7/8* morphants (Figure 2D lower panel; 2G). In contrast, ectopic expression of *e2f7/8* mRNA resulted in increased number of both, PLs and vISVs, without any TD phenotype (Figure 2D–G). Together these results suggest that *e2f7/8* regulate the number of cells emerging from the PCV during venous sprouting.

### Ectopic Expression of *ccbe1* Partially Restored Venous Sprouting in *e2f7/8* Deficient Zebrafish

Given that Ccbe1 stimulates lymphangiogenesis and its expression is regulated by atypical E2Fs, we reasoned that the lymphatic phenotype in *e2f7/8* morphants is a result of decreased *ccbe1* expression. To test this hypothesis, we co-injected *ccbe1* mRNA in *e2f7/8* morphants. Ectopic expression of *ccbe1* alone resulted in an increased numbers of PLs at the HM at 52 hpf (Figure 2H and I). In addition, the increased number of PLs did not affect the formation of the TD 5 dpf (Figure 2H–J). Co-injecting *ccbe1* mRNA in *e2f7/8* morphants resulted in a partial rescue of the number of PLs at 52 hpf, however, these PLs showed an abnormal morphology (Figure 2H and I). To this extent, it appeared that the majority of PLs connected to ISVs, however at ectopic positions and without generating functional venous connections (Figure 2H white arrows). The TD defects of *e2f7/8* morphants were not rescued by ectopic expression of *ccbe1*, possibly due to the early connection of the PLs to the ISVs (Figure 2H and J). Furthermore, we show that ectopic overexpression of *flt4* does not phenocopy the lymphangiogenesis defects of *e2f7/8* morphants (Figure S2A). Together, these findings suggest that the lymphatic phenotype in *e2f7/8* morphants is likely the result of decreased expression of *ccbe1*.

### Induction of E2f7/8 Restored Ccbe1-dependent Venous Sprouting and Lymphangiogenesis

**Figure 3:**
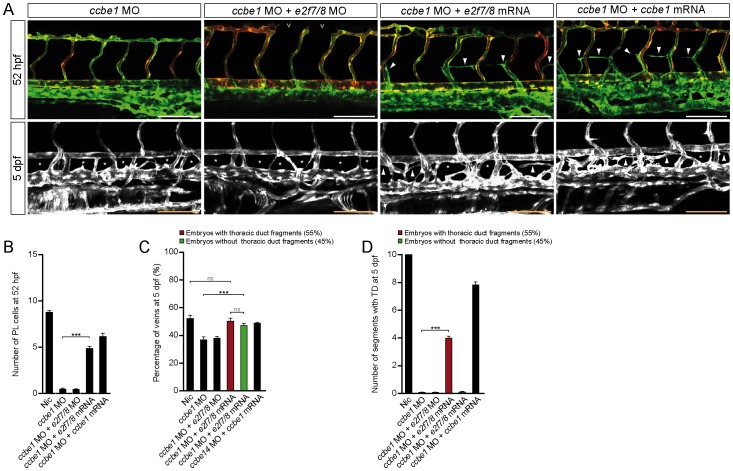
E2f7/8 rescued Ccbe1dependent lymphangiogenesis phenotype. A, Representative images of *Tg(fli1a:gfp;flt1^enh^:rfp*) un-injected control embryos (nic) or embryos injected with *e2f7/8* MOs or mRNA. B, C, D, Quantification of the indicated parameters. Concentrations: *e2f7/8* MOs (10 ng each); *ccb1* MO (5 ng); *e2f7/8* mRNA (100 pg each); *ccbe1* mRNA (100 pg).Open arrow heads indicate in (A; upper panel) missing dorsal longitudinal anastomotic vessels. Closed arrow heads indicate (upper panel in A) PLs or (lower panel, A) presence of the TD. All scale bars are 100 µm. Stars indicate missing TD fragments. Data presented as the average (±s.e.m.) compared to the control condition in three independent experiments (*** *P*<0.001). At least n = 150 embryos per condition in three independent experiments were used for A–D.

Considering that *e2f7/8* mRNA induced *ccbe1* expression (Figure 2A,B), we hypothesized that *e2f7/8* are able to rescue the *ccbe1* morphant phenotype, characterized by reduced venous sprouting, which consequently leads to a decreased number of veins, absence of PLs and loss of TD (Figure 3A–D) [13]. Consistent with the data that loss of E2F7/8 results in a decreased expression of CCBE1, injection of *e2f7/8* MOs together with *ccbe1* MOs resulted in no apparent improvement of the *ccbe1* MO induced phenotype (Figure 3A–D). However, as hypothesized, co-injecting *e2f7/8* mRNA or *ccbe1* mRNA in *ccbe1* morphants resulted in partial restoration of the number of PLs at the HM and reappearance of TD fragments in 55% of the morphants (Figure 3A,B,D). Although we could only rescue 55% of the embryos co-injected with *ccbe1* MO and *e2f7/8* mRNA on the level of the TD, we found in almost all embryos a rescue in the number of veins to the same extent as with *ccbe1* mRNA, (Figure 3C). These findings suggest that E2F7/8 promote venous sprouting and lymphangiogenesis through transcriptional activation of *ccbe1*.

### E2f7/8 Modulate Flt4-dependent Venous Sprouting, Lymphangiogenesis, and arterial Hyperbranching

**Figure 4:**
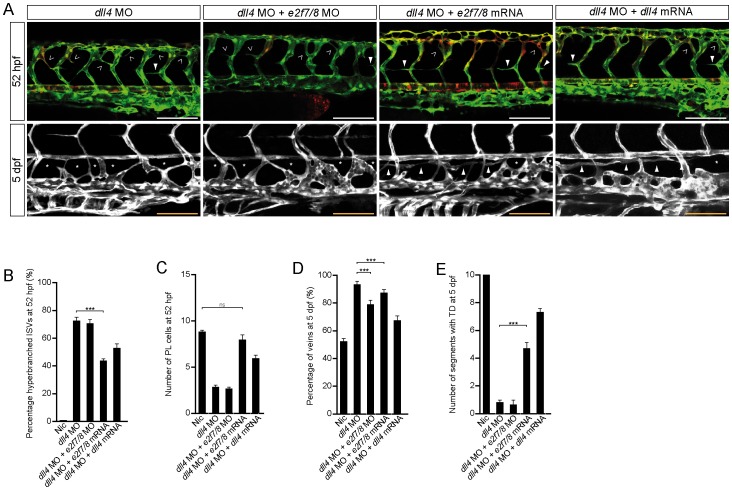
E2f7/8 rescued Flt4-dependent lymphangiogenesis phenotypes. A, Representative images of *Tg(fli1a:gfp;flt1^enh^:rfp*) un-injected control embryos (nic) or embryos injected with *e2f7/8* MOs or mRNA. B, C, D, E Quantification of the indicated parameters. Concentrations: *e2f7/8* MOs (10 ng each); *dll4* MO (3 ng); *e2f7/8* mRNA (100 pg each); *dll4* mRNA (100 pg).Open arrow heads indicate in (A; upper panel) hyper-branching of intersegmental vessels. Closed arrow heads indicate (upper panel in A) PLs or (lower panel, A) presence of the TD. All scale bars are 100 µm. Stars indicate missing TD fragments. Data presented as the average (±s.e.m.) compared to the control condition in three independent experiments (*** *P*<0.001). At least n = 150 embryos per condition in three independent experiments were used for A–E.

During venous sprouting, VegfC signaling specifically drives the budding of (venous) ECs from the PCV, whereas ECs from the DA and aISVs seem to be non-responsive to VegfC. Recently, it has been shown that Dll4, which is expressed in the DA and aISVs, maintains arterial ECs in a quiescent state during venous sprouting. More specific, it was reported that Dll4 represses VegfC-Flt4 signaling in arterial cells, without affecting *flt4* and *vegfC* mRNA levels and loss of Dll4 therefore resulted in hyperbranching of aISVs (Figure 4A open arrowheads) [2]. We used this hyperbranching phenotype during the onset of venous sprouting in *dll4* morphants to assess the transcriptional repression of E2f7/8 on Flt4 signaling *in vivo.* In line with our finding that *e2f7/8* mRNA injections leads to decreased *flt4* levels, we found that ectopic expression of *e2f7/8* mRNA partially suppressed the hyperbranching phenotype in *dll4* morphants (Figure 4A,B). Next, we evaluated whether this suppression of hyperbranching by E2f7/8 was specifically regulated through modulation of *flt4* expression. First, we assessed if ectopically expressed *flt4* was able to induce a hyperbranching phenotype. At 52 hpf we sporadically found hyperbranching of ISVs in *flt4* mRNA injected embryos (Figure S2A and B). We concluded that the suppression of Dll4 on Flt4 signaling was robust enough to compensate for the increased Flt4 signaling in *flt4* mRNA injected embryos. To sensitize ISVs for increased Flt4 signaling, we injected low amounts of *dll4* (low) morpholino and found a dramatic increase in hyperbranching when *flt4* mRNA was co-injected in *dll4* (low) morphants (Figure S2A and B). These findings provide strong evidence that Flt4 signaling promotes the hyperbranching phenotype in *dll4* morphants. This increased hyperbranching phenotype in *dll4* (low) MO, induced by *flt4* mRNA, was suppressed by co-injecting *e2f7/8* mRNA. These studies suggest that ectopic expression of *e2f7/8* represses endogenous *flt4* expression and thereby reduces the total *flt4* levels in *flt4* mRNA and *dll4* MO (low) injected embryos, and consequently inhibits hyperbranching. Together, these results indicate that Dll4 modulates Flt4 signaling in a transcriptional independent manner [2], while E2f7/8 likely regulate Flt4 signaling at the transcriptional level. Furthermore, analysis of *dll4* expression in *e2f7/8* mRNA injected embryos by *in situ* hybridization, showed a decrease in *dll4* mRNA (data not shown), excluding the possibility that E2f7/8-mediated suppression of the hyperbranching phenotype of *dll4* morphants occurs through upregulation of *dll4* expression.

In addition to the hyperbranching phenotype, *dll4* morphants also displayed an almost complete absence of PLs and loss of TD (Figure 4A,C,D,E) [2]. Moreover, ISVs of *dll4* morphants have a predominantly venous identity, suggesting that venous sprouting is not completely lost, but that cells budding from the PCV are pre-dominantly programmed to connect to aISVs rather than migrating to the HM. To investigate if these phenotypes were also due to loss of *dll4* suppression on Flt4 signaling and whether E2f7/8 are able to rescue these phenotypes by modulating *flt4* expression, we first co-injected *dll4* MO and *e2f7/8* MOs. In line with the results that KD of *dll4* leads to the loss of repression of VegfC-Flt4 signaling and loss of *e2f7/8* would only lead to a further increase of signaling by increased amounts of *flt4,* we found no improvement of the phenotype in *e2f7/8* and *dll4* double-morphants (Figure 4B–D). In contrast, ectopic expression of either *e2f7/8* mRNA or *dll4* mRNA in *dll4* morphants, increased the number of PLs to almost *wild-type* levels. Moreover, the TD was partially restored at 5 dpf. As ectopic expression of *e2f7/8* also consequently leads to increased expression of *ccbe1*, we checked whether the rescue of the TD in *dll4* morphants injected with *e2f7/8* mRNA was specifically due to repression of *flt4* expression and not due to increased *ccbe1* expression. To this extent, we injected *ccbe1* mRNA in *dll4* morphants and observed a minor increase in PLs at 52 hpf whereas at 5 dpf the TD was absent (Figure S2C–F). In addition, the morphology of the PLs in embryos injected with *ccbe1* mRNA and *dll4* MO (Figure S2C; arrows) showed great similarity with PLs in embryos injected with *ccbe1* mRNA and *e2f7/8* MOs (Figure 2H; arrows). These results suggest that the amount of Flt4 signaling might determine whether venous sprouts contribute to the vascular system or will migrate further to the HM and contribute to the lymphatic system. As mentioned above, the vasculature of *dll4* morphants consists out of almost 90% vISVs (Figure 4A,D) and in line with our previous data, injection of *e2f7/8* mRNA or *dll4* mRNA in *dll4* morphants reduced the number of veins (Figure 4D). However, injection of *e2f7/8* MOs in *dll4* morphants also significantly reduced the number of veins in *dll4* morphants (Figure 4D). In regard with our previous data, loss of E2f7/8 in *dll4* morphants leads, next to the increased expression of *flt4*, also to a decreased expression of *ccbe1* (Figure 2A,B), resulting in reduced venous sprouting and consequently to a lower number of vISVs.

Together, these data provide strong evidence that E2f7/8 can compensate for the loss of repression on Flt4 signaling in *dll4* morphants by transcriptional repression of *flt4*, thereby leading to the partial rescue of the hyperbranching phenotype, the arterial-venous patterning defects and the impaired formation of PLs and TD.

## Discussion

In this study, we show that two important players of lymphangiogenesis, Flt4 and Ccbe1, are directly regulated by atypical E2Fs. We demonstrate that inactivation of E2f7/8 in zebrafish impairs venous sprouting and lymphangiogenesis accompanied by deregulated expression of *ccbe1* and *flt4*. Moreover, we show that E2f7/8 induction can rescue lymphangiogenesis defects caused by loss of Ccbe1 or by enhanced Flt4 signaling due to the loss of Dll4. From these findings we conclude, that E2f7/8 are required for lymphangiogenesis through fine-tuning Ccbe1 and Flt4 expression.

We recently showed that ablation of *e2f7/8* in zebrafish resulted in a *vegfAa* dependent angiogenesis phenotype [11]. However, several studies indicated that venous sprouting and, thereby, lymphangiogenesis is a VegfA independent process in zebrafish. It was shown that VegfA dependent phosphorylation of Vegf receptor 2 (KDR/Flk-1) acts mainly through downstream signaling via the phospholipase C-γ (PLC-γ) - protein kinase C (PKC) - Raf-mitogen activated protein (MAP) kinase pathway [18], [19]. Zebrafish mutant for PLC-γ or injected with PLC-γ morpholino oligomers showed specific defects in the formation of arteries, but not veins or parachordal lymphangioblasts [13], [20]. These findings indicate that the lymphatic phenotype observed in *e2f7/8* deficient zebrafish is unlikely due to decreased *vegfAa* expression.

Recently, it was shown that CCBE1 enhances the biological effect of VEGFC on lymphangiogenesis [5], [21]. This effect is most likely mediated through VEGFR3 and raises the question why E2F7/8 regulates CCBE1 and VEGFR3 in opposing ways. Our expression data of the atypical E2Fs, CCBE1 and FLT4 in different cell lines revealed that the atypical E2Fs are almost equally expressed between these cell lines while CCBE1 (mesenchymal cells) and FLT4 (endothelial cells) show a cell type specific expression pattern (Figure S1B). Moreover, this cell type specific expression matches with the expression of E2F7/8, FLT4 and CCBE1 *in vivo* ([2], [5], [6], [13]. Furthermore, our experiments show that the amount of Flt4 signaling, either modulated on the level of expression or on the level of signaling output, *in vivo* is of crucial importance for proper lymphangiogenesis in zebrafish embryos. This observation suggests that genes involved in regulating Flt4 signaling need a precise and timely regulation. Our results suggest that E2F7/8 do not function as a ON/OFF switch for expression of *ccbe1* and *flt4*. Instead, our findings support a model where E2F7/8 fine-tune the expression levels of *ccbe1* and *flt4* in cell-type specific manner and provide thereby a mechanism that ensures the right amount of Flt4 signaling during lymphangiogenesis. In this model, the transcriptional effect of the atypical E2Fs on the promoter of their target genes depends on their interaction with co-factors and, moreover, is cell type specific. To this extent, several reports show that E2Fs recruit co-factors that determines their transcriptional potency [22]–[24]. Recently, we showed that E2F7 needs to cooperate with HIF1 to induce VEGFA transcription [11]. In this specific case, HIF1 not only determines the transcriptional activity of E2F7, but also provides an additional way to interact with the DNA through HIF binding sites. Thus, we suggest that the contrasting modulation of Ccbe1 and Flt4 by E2f7/8 might be achieved by the recruitment of additional factors, which display a tissue specific expression. The overlap in expression for *hif1α* [25], *ccbe1* [13] and *e2f7/8* [11] in zebrafish embryos might be Illustrative for this hypothesis.

The dual role of atypical E2Fs in regulating cell cycle and lymphangiogenesis is particularly interesting in the context of cancer. Atypical E2Fs are highly expressed in many types of tumors (Oncomine). Furthermore, dissemination of tumor cells often occurs via the lymphatic system and can be blocked by interfering with FLT4 signaling [26], [27]. Thus, it would be of great interest to investigate whether E2F7/8 not only control CCBE1 and FLT4 during developmental lymphangiogenesis but also during tumor dissemination.

Our previous finding that *e2f7/8* regulate angiogenesis [11], provided strong evidence that atypical E2fs possess functions that reach beyond cell cycle control. In support, we show here that E2f7/8 regulate Ccbe1 and Flt4, two indispensable genes for lymphangiogenesis *in vivo*.

## Methods

### Ethics Statements

Animal experiments were performed in accordance with the rules of the Animal Experimentation Committee of the Royal Netherlands Academy of Arts and Sciences (DEC).

### Zebrafish

All zebrafish strains were maintained in the Hubrecht Institute (Utrecht Medical Center, Netherlands) under standard husbandry conditions. Transgenic lines used *Tg(fli1a:gfp*)^y1^ [28], *Tg(flt1^enh^:rfp)* [17]. The transgenic reporter line *Tg(flt4:YFP)* was generated from BAC DKEY-58G10 using standard methods [17] and will be described in detail elsewhere.

### Quantification

Parachordal lymphangioblasts [13], [17] and thoracic duct were quantified in zebrafish embryos as follows: the space between two intersegmental vessels was considered as 1 segment. 10 segments anterior from the end of the yolk sac extension were quantified at the indicated time points.

### Micro-array Analysis

Data from Gene Expression Omnibus (GEO) database number GSE30488 was analyzed by FlexArray [29]. All significant genes associated with AmiGO gene ontology lists, angiogenesis (GO:0001525) were extracted and analyzed for E2F binding elements within their proximal promoters with DAVID (http://david.abcc.ncifcrf.gov/ [30], [31]). Additionally, genes with E2F binding elements that showed a deregulation (up or down) were further analyzed for their association with GO term lymphangiogenesis (GO:0001946). GO terms were retrieved from (http://www.geneontology.org/ [32]). Promoter analysis was performed with Consite (http://asp.ii.uib.no:8090/cgi-bin/CONSITE/consite/).

### Morpholino

The following morpholino oligonucleotides (Genetools) were used. *E2f7* splice morpholino targeting exon 2–intron 2–3: 5′-ATAAAGTACGATTATCCAAATGCAC-3′ [11]; *e2f8* splice morpholino targeting exon 2-intron 2–3: 5′-CTCACAGGTATCCGAAAAAGTCATT-3′ [11]; *dll4* splice morpholino targeting exon 4– intron 4–5: 5′- TGATCTCTGATTGCTTACGTTCTTC-3′ [2]; *ccbe1* ATG morpholino: 5′- CGGGTAGATCATTTCAGACACTCTG-3′ [13]. Concentration of morpholinos used are noted in the figure legends.

### Imaging

Embryos were mounted in 0.5–1% low melting point agarose (Invtrogen) dissolved in E3 buffer (5 mM NaCl, 0.17 mM KCl, 0.33 mM CaCl_2_, 0.33 mM MgSO_4_) on a culture dish with a glass cover slip replacing the bottom. Imaging was performed with a Leica SP2 confocal microscope (Leica Microsystems) using a 10× or 20× objective with digital zoom.

### Quantification flt4:YFP Reporter Line

For all experiments the same male and female *flt4*:YFP fish were crossed and offsprings were either injected with *e2f7/8* MO or uninjected. For each condition 30 embryos were imaged by confocal microscopy with the same settings in three independent experiments. Images were processed with image J. First the image was converted to a binary image. Second, all black pixels were quantified by imageJ and used for statistical analysis (Mann–Whitney *U*-test).

### Plasmid Constructs

*E2f7* [11] and *e2f8* [11] pCS^2+^ plasmids were linearized with *NotI a*nd mRNA was synthesized using the SP6 RNA polymerase and the mMachine kit (Ambion).

### In situ Hybridization

In situ hybridization was performed as previously described [33]. The *ccbe1* [13] *and flt4* [34] probes have been described previously. Probes were synthesized by *in vitro* transcription using T7 RNA polymerase (Promega).

### Cell Culture, Overexpression and siRNA Transfection

Cervical cancer (HeLa) cell lines were cultured in DMEM (Invitrogen, 41966-052) supplemented with 10% FBS (Lonza, DE14-802F). Lymphatic endothelial cells (LECs) were cultured in supplemented EGM-2 medium (Lonnza). Cells were transfected as specified by the manufacturer using 5 µl/p60 dish (Greiner) Lipofectamine 2000 (Invitrogen) with the following concentrations of siRNA: single transfections had a final concentration of 25 nM siRNA, double transfection had a final concentration of 50 nM siRNAs (2×25 nM). In case of single transfection the Scrambled siRNA concentration was 25 nM, while in case of double transfection the concentration of the Scrambled siRNA was 50 nM. Cell were grown for 24 hours. Alternatively, overexpression was induced by adding 0.2 µg ml^−1^ doxycycline (Sigma) to the cell culture medium, treatment duration was 12 hours. After incubation of siRNAs or doxycycline with the indicated duration, cells were washed with PBS and trypsinized (lonza; cc-5012), pelleted by centrifugation (2600×g, 2′, 4°C), snap freezed in liquid nitrogen and stored in −80°C for RNA or protein isolation. Protein was isolated by disrupting cells in lysis buffer (0.05 M sodium phosphate pH 7.3, 0.3 M NaCl, 0.1% NP40, 10% Glycerol) supplemented with protease inhibitors (Roche). Total RNA was extracted according to manufacturers’ instructions using the RNeasy Mini Kit (Qiagen, cat #74106).

RNAi used in this study: scrambled siRNA #2 (D-001210-02, Thermo Scientific). hE2F7 (HSS175354, Invitrogen), hE2F8 (HSS128760, Invitrogen).

### SDS-PAGE, Western Blot and Protein Quantification

Cells were harvested (as described under siRNA transfection). Cell lysates were subjected to standard ECL reagents as described by the manufacturer (GE Healthcare, RPN2106). Used antibodies: E2F1 (Santa Cruz Biotechnology, sc-193) E2F7 (Santa Cruz Biotechnology, sc-66870), E2F8 (Abcam, AB109596), FLT4 (Santa Cruz Biotechnology; sc-321), ERK (Santa Cruz Biotechnology; sc-94), pERK (Santa Cruz Biotechnology; sc-7383), Mouse IgG HRP-linked whole Ab (GE Healthcare, NA931), Rabbit IgG HRP-linked whole Ab (GE Healthcare, NA934).

Relative protein levels were determined with gel analyzer in ImageJ (http://rsbweb.nih.gov/ij/). Protein loading was equalized with TUBULIN and fold changes were calculated.

### Chromatin Immunoprecipitation (ChIP)

ChIP was performed according the EZ ChIP protocol (Upstate, 17–371) using protein G agarose beads (Milipore, 16–266) coated overnight in 0,1% BSA (Sigma, A3294). The following antibodies were used: E2F7 (Santa Cruz Biotechnology; sc-66870) and E2F8 (Abcam, AB109596). De-crosslinked DNA was purified over a column (Qiagen, 28106) and eluted in 50 µl H_2_O of which 2 µl was used for quantitative PCR.

### RNA Isolation, cDNA Synthesis and Quantitative PCR

cDNA was synthesized with random hexamer primers according to manufacturers’ instructions (Fermentas, cat#K1622). Quantitative PCR was performed on a MyiQ cycler (Biorad) using SYBRgreen chemistry (Biorad). In our *in vitro* studies two reference genes were used (ACTB, RPS18) and for zebrafish samples three reference genes were used (TBP, EF1α, β Actin). MIQE standards were applied to our protocols [35]. The sequences of all primers used in this manuscript are shown in Table 1.

**Table 1:** Primers used for ChIP and qPCR.

Primer	Technique; Species	Forward*	Reversed*
CCBE1	qPCR; Human	TACCGATATGACCGGGAGAG	AGCTGCCCAAGGTATTGATG
FLT4	qPCR; Human	GAGACAAGGACAGCGAGGAC	CTGTGTCGTTGGCATGTACC
E2F7	qPCR; Human	CTCCTGTGCCAGAAGTTTC	CATAGATGCGTCTCCTTTCC
E2F8	qPCR; Human	AATATCGTGTTGGCAGAGATCC	AGGTTGGCTGTCGGTGTC
E2F1	qPCR; Human	GACCACCTGATGAATATCTG	TGCTACGAAGGTCCTGAC
VEGFA	qPCR; Human	ACCTCCACCATGCCAAGTG	TCTCGATTGGATGGCAGTAG
ACTB	qPCR; Human	GATCGGCGGCTCCATCCTG	GACTCGTCATACTCCTGCTTGC
RSP18	qPCR; Human	AGTTCCAGCATATTTTGCGAG	CTCTTGGTGAGGTCAATGTC
CCBE1	Chip; Human	CCCTCCTCCGTTTTCTTGTT	TTGTCCTGAGCGGCTTTAAT
FLT4	Chip; Human	TTGGAGAGAGCTGGTAGTGG	CCTGTAATCCCAGCTTCTCG
E2F1	ChIP; Human	AGGAACCGCCGCCGTTGTTCCCGT	CTGCCTGCAAAGTCCCGGCCACTT
E2F1 control	ChIP; Human	CGCCCAGACGCCACTTCATC	TTCATTCCCTCACTCATTCAACAA
Ccbe1	qPCR; Zebrafish	AATCGCAACGACGAAGTACC	CCGGCACACACATCATAATC
Flt4	qPCR; Zebrafish	TGCACCAGTATGCCACATTT	TGCTTCCATTGCTTTGACTG
E2f1	qPCR; Zebrafish	ACGCATCTACGACATCACCA	CTCCGTCAGCTCAGAAACCT
TBP	qPCR; Zebrafish	TCACCCCTATGACGCCTATC	CAAGTTGCACCCCAAGTTT
EF1α	qPCR; Zebrafish	GATTGTTGCTGGTGGTGTTG	TGTATGCGCTGACTTCCTTG
B-Actin	qPCR; Zebrafish	CGTCTGGATCTAGCTGGTCGTGA	CAATTTCTCTTTCGGCTGTGGTG

### Statistical Analysis

For statistical analysis of two groups, unpaired *t*-test, or in case of unequal variances, Mann–Whitney *U*-test were used. For statistical analysis of multiple groups, one-way ANOVA, or in case of unequal variances, Kruskal–Wallis test was used. Dunns *post hoc* test were used to compare between selected groups. *P*-values<0.05 were considered significant. Statistical analysis was performed using SPSS 20 (IBM).

## Supporting Information

Figure S1 **E2F7/8 directly regulate CCBE1 and FLT4 expression.** S1A, protein and mRNA expression of indicated genes in HeLa cells treated with Scrambled, *E2F1*, *E2F7, E2F8* or *E2F7/8* siRNAs. S2B, Relative expression of indicated gene in HeLa, HUVECs, MEFs and LECs. Additional, *in silico* analysis of the trimethylated Lys4 and Lys27 mark on histone H3 in HeLa, HUVECs and normal human lung fibroblasts (NHLF). S1C, indicated mRNA levels in Cre inducible *E2f7^loxP/loxP^E2f8^loxP/loxP^* MEFs treated with tamoxifen (0.2 µg /ml) for 24 hours and E2F7/8 siRNA treated HUVECs and LECs. Data presented as the average (±s.e.m.) compared to the control condition in two independent experiments.
(TIF)

Figure S2 **Hyperbranching and venous sprouting is dependent on proper Flt4 signaling.** S2A Lateral images and quantification of *Tg(fli1a:gfp;flt1^enh^:rfp*) embryos treated as indicated and imaged at 52 hpf. S2C Lateral images and quantification of *Tg(fli1a:gfp;flt1^enh^:rfp*) embryos treated as indicated and imaged at 52 hpf. S2D, S2E, S2F, quantification of the indicated parameters. Concentrations: *dll4* MO (low 1.5 ng in S2A and 3 ng in S2C–F); *e2f7/8* mRNA (100 pg each); *flt4* mRNA (100 pg); *ccbe1* mRNA (100 pg). Arrows depict PLs that have connected to ISVs (S2C). Closed arrow heads indicate (upper panel in S2C) PLs or (lower panel, S2C) presence of the TD. Open arrowheads indicate hyperbranching ISVs. All scale bars are 100 µm. Stars indicate missing TD fragments. Data presented as the average (±s.e.m.) compared to the control condition in three independent experiments (*** *P*<0.001). At least n = 150 embryos per condition in three independent experiments were used for S2A–S2F.
(TIF)

## References

[1] Tammela T, Alitalo K (2010) Lymphangiogenesis: Molecular mechanisms and future promise Cell. 140: 460–476. 10.1016/j.cell.2010.01.045 20178740

[2] Hogan BM, Herpers R, Witte M, Helotera H, Alitalo K, et al (2009) Vegfc/Flt4 signalling is suppressed by Dll4 in developing zebrafish intersegmental arteries Development. 136: 4001–4009. 10.1242/dev.039990 19906867

[3] Karkkainen MJ, Haiko P, Sainio K, Partanen J, Taipale J, et al (2004) Vascular endothelial growth factor C is required for sprouting of the first lymphatic vessels from embryonic veins Nat Immunol. 5: 74–80. 10.1038/ni1013 14634646

[4] Schulte-Merker S, Sabine A, Petrova TV (2011) Lymphatic vascular morphogenesis in development, physiology, and disease J Cell Biol. 193: 607–618. 10.1083/jcb.201012094 PMC3166860 21576390

[5] Bos FL, Caunt M, Peterson-Maduro J, Planas-Paz L, Kowalski J, et al (2011) CCBE1 is essential for mammalian lymphatic vascular development and enhances the lymphangiogenic effect of vascular endothelial growth factor-C in vivo Circ Res. 109: 486–491. 10.1161/CIRCRESAHA.111.250738 21778431

[6] Li J, Ran C, Li E, Gordon F, Comstock G, et al (2008) Synergistic function of E2F7 and E2F8 is essential for cell survival and embryonic development. Dev Cell 14: 62–75. 18194653 10.1016/j.devcel.2007.10.017 PMC2253677

[7] de Bruin A, Maiti B, Jakoi L, Timmers C, Buerki R, et al (2003) Identification and characterization of E2F7, a novel mammalian E2F family member capable of blocking cellular proliferation J Biol Chem. 278: 42041–42049. 10.1074/jbc.M308105200 12893818

[8] Maiti B, Li J, de Bruin A, Gordon F, Timmers C, et al (2005) Cloning and characterization of mouse E2F8, a novel mammalian E2F family member capable of blocking cellular proliferation J Biol Chem. 280: 18211–18220. 10.1074/jbc.M501410200 15722552

[9] Pandit SK, Westendorp B, Nantasanti S, van Liere E, Tooten PCJ, et al (2012) E2F8 is essential for polyploidization in mammalian cells Nat Cell Biol. 14: 1181–1191. 10.1038/ncb2585 23064264

[10] Westendorp B, Mokry M, Groot Koerkamp MJ, Holstege FC, Cuppen E, et al (2012) E2F7 represses a network of oscillating cell cycle genes to control S-phase progression Nucleic Acids Res. 40(8): 3511–23. 10.1093/nar/gkr1203 PMC3333892 22180533

[11] Weijts BGMW, Bakker WJ, Cornelissen PWA, Liang K, Schaftenaar FH, et al (2012) E2F7 and E2F8 promote angiogenesis through transcriptional activation of VEGFA in cooperation with HIF1 EMBO J. 31: 3871–3884. 10.1038/emboj.2012.231 PMC3463843 22903062

[12] Ouseph MM, Li J, Chen HZ, Pecot T, Wenzel P, et al (2012) Atypical E2F repressors and activators coordinate placental development Dev Cell. 22: 849–862. 10.1016/j.devcel.2012.01.013 PMC3483796 22516201

[13] Hogan BM, Bos FL, Bussmann J, Witte M, Chi NC, et al (2009) Ccbe1 is required for embryonic lymphangiogenesis and venous sprouting Nat Genet. 41: 396–398. 10.1038/ng.321 19287381

[14] Kazenwadel J, Secker GA, Betterman KL, Harvey NL (2012) In vitro assays using primary embryonic mouse lymphatic endothelial cells uncover key roles for FGFR1 signalling in lymphangiogenesis PLoS One. 7: e40497. 10.1371/journal.pone.0040497 PMC3391274 22792354

[15] Ruthenburg AJ, Li H, Patel DJ, David Allis C (2007) Multivalent engagement of chromatin modifications by linked binding modules Nature Reviews Molecular Cell Biology. 8: 983–994. 10.1038/nrm2298 PMC4690530 18037899

[16] Bernstein BE, Mikkelsen TS, Xie X, Kamal M, Huebert DJ, et al (2006) A bivalent chromatin structure marks key developmental genes in embryonic stem cells. Cell 125: 315–326. 16630819 10.1016/j.cell.2006.02.041

[17] Bussmann J, Bos FL, Urasaki A, Kawakami K, Duckers HJ, et al (2010) Arteries provide essential guidance cues for lymphatic endothelial cells in the zebrafish trunk Development. 137: 2653–2657. 10.1242/dev.048207 20610484

[18] Takahashi T, Yamaguchi S, Chida K, Shibuya M (2001) A single autophosphorylation site on KDR/Flk-1 is essential for VEGF-A-dependent activation of PLC-gamma and DNA synthesis in vascular endothelial cells EMBO J. 20: 2768–2778. 10.1093/emboj/20.11.2768 PMC125481 11387210

[19] Takahashi T, Shibuya M (1997) The 230 kDa mature form of KDR/Flk-1 (VEGF receptor-2) activates the PLC-gamma pathway and partially induces mitotic signals in NIH3T3 fibroblasts Oncogene. 14: 2079–2089. 10.1038/sj.onc.1201047 9160888

[20] Lawson ND, Mugford JW, Diamond BA, Weinstein BM (2003) Phospholipase C gamma-1 is required downstream of vascular endothelial growth factor during arterial development Genes Dev. 17: 1346–1351. 10.1101/gad.1072203 PMC196067 12782653

[21] Hagerling R, Pollmann C, Andreas M, Schmidt C, Nurmi H, et al (2013) A novel multistep mechanism for initial lymphangiogenesis in mouse embryos based on ultramicroscopy EMBO J. 32: 629–644. 10.1038/emboj.2012.340 PMC3590982 23299940

[22] Ogawa H, Ishiguro K, Gaubatz S, Livingston DM, Nakatani Y (2002) A complex with chromatin modifiers that occupies E2F- and myc-responsive genes in G0 cells Science. 296: 1132–1136. 10.1126/science.1069861 12004135

[23] Korenjak M, Anderssen E, Ramaswamy S, Whetstine JR, Dyson NJ (2012) RBF binding to both canonical E2F targets and noncanonical targets depends on functional dE2F/dDP complexes Mol Cell Biol. 32: 4375–4387. 10.1128/MCB.00536-12 PMC3486151 22927638

[24] Lu Z, Luo RZ, Peng H, Huang M, Nishmoto A, et al (2006) E2F-HDAC complexes negatively regulate the tumor suppressor gene ARHI in breast cancer Oncogene. 25: 230–239. 10.1038/sj.onc.1209025 16158053

[25] Rojas DA, Perez-Munizaga DA, Centanin L, Antonelli M, Wappner P, et al (2007) Cloning of hif-1alpha and hif-2alpha and mRNA expression pattern during development in zebrafish Gene Expr Patterns. 7: 339–345. 10.1016/j.modgep.2006.08.002 16997637

[26] Roberts N, Kloos B, Cassella M, Podgrabinska S, Persaud K, et al (2006) Inhibition of VEGFR-3 activation with the antagonistic antibody more potently suppresses lymph node and distant metastases than inactivation of VEGFR-2 Cancer Res. 66: 2650–2657. 10.1158/0008-5472.CAN-05-1843 16510584

[27] Laakkonen P, Waltari M, Holopainen T, Takahashi T, Pytowski B, et al (2007) Vascular endothelial growth factor receptor 3 is involved in tumor angiogenesis and growth Cancer Res. 67: 593–599. 10.1158/0008-5472.CAN-06-3567 17234768

[28] Lawson ND, Weinstein BM (2002) In vivo imaging of embryonic vascular development using transgenic zebrafish. Dev Biol 248: 307–318. 12167406 10.1006/dbio.2002.0711

[29] Blazejczyk M, Miron M, Nadon R (2007) FlexArray: A statistical data analysis software for gene expression microarrays.

[30] Huang da W, Sherman BT, Lempicki RA (2009) Systematic and integrative analysis of large gene lists using DAVID bioinformatics resources Nat Protoc. 4: 44–57. 10.1038/nprot.2008.211 19131956

[31] Huang da W, Sherman BT, Lempicki RA (2009) Bioinformatics enrichment tools: Paths toward the comprehensive functional analysis of large gene lists Nucleic Acids Res. 37: 1–13. 10.1093/nar/gkn923 PMC2615629 19033363

[32] Ashburner M, Ball CA, Blake JA, Botstein D, Butler H, et al (2000) Gene ontology: Tool for the unification of biology. the gene ontology consortium Nat Genet 25: 25–29. 10802651 10.1038/75556 PMC3037419

[33] Bussmann J, Bakkers J, Schulte-Merker S (2007) Early endocardial morphogenesis requires Scl/Tal1 PLoS Genet. 3: e140. 10.1371/journal.pgen.0030140 PMC1950955 17722983

[34] Thompson MA, Ransom DG, Pratt SJ, MacLennan H, Kieran MW, et al (1998) The cloche and spadetail genes differentially affect hematopoiesis and vasculogenesis Dev Biol. 197: 248–269. 10.1006/dbio.1998.8887 9630750

[35] Bustin SA, Benes V, Garson JA, Hellemans J, Huggett J, et al (2009) The MIQE guidelines: Minimum information for publication of quantitative real-time PCR experiments Clin Chem. 55: 611–622. 10.1373/clinchem.2008.112797 19246619

