# Correction: Atypical E2fs Control Lymphangiogenesis through Transcriptional Regulation of Ccbe1 and Flt4

**DOI:** 10.1371/annotation/25c7c486-9e68-4df9-af01-2fe1c5d1a2d4

**Published:** 2013-09-17

**Authors:** Bart G. M. W. Weijts, Andreas van Impel, Stefan Schulte-Merker, Alain de Bruin

There was information omitted from the Funding statement.

The correct Funding statement should read: This work was supported by the Netherlands Organization for Scientific Research (NWO-ALW 834.11.002 and 036.002.598). The funders had no role in study design, data collection and analysis, decision to publish, or preparation of the manuscript. 

