# Supplementary figures and images for: Atypical E2fs Control Lymphangiogenesis through Transcriptional Regulation of Ccbe1 and Flt4
Source: PLoS One. 2013 Sep 12;8(9):e73693. doi: 10.1371/journal.pone.0073693 (PMC3771987; doi:10.1371/journal.pone.0073693)

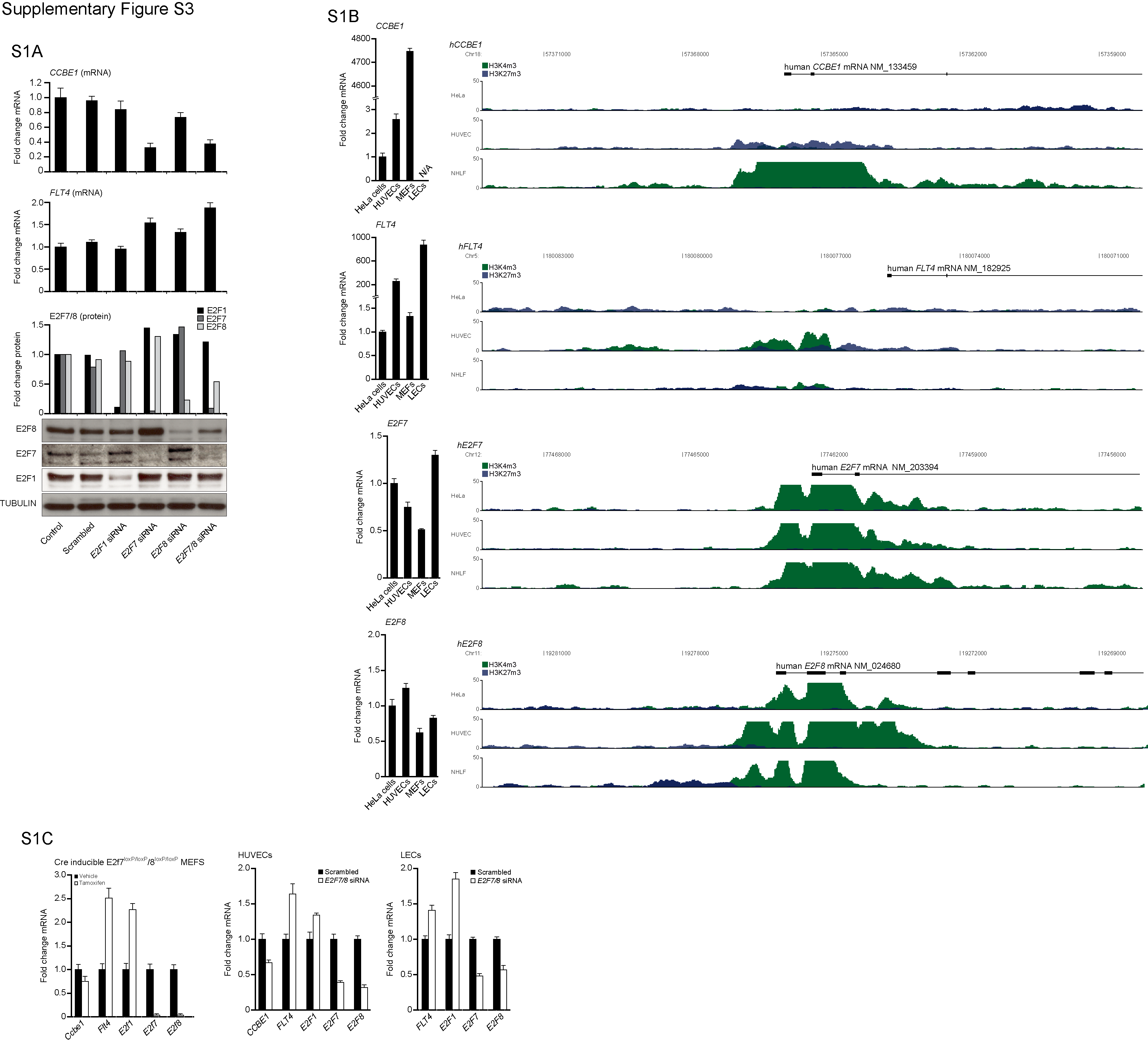

Supplement: Figure S1 — E2F7/8 directly regulate CCBE1 and FLT4 expression. S1A, protein and mRNA expression of indicated genes in HeLa cells treated with Scrambled, E2F1, E2F7, E2F8 or E2F7/8 siRNAs. S2B, Relative expression of indicated gene in HeLa, HUVECs, MEFs and LECs. Additional, in silico analysis of the trimethylated Lys4 and Lys27 mark on histone H3 in HeLa, HUVECs and normal human lung fibroblasts (NHLF). S1C, indicated mRNA levels in Cre inducible E2f7loxP/loxPE2f8loxP/loxP MEFs treated with tamoxifen (0.2 µg /ml) for 24 hours and E2F7/8 siRNA treated HUVECs and LECs. Data presented as the average (±s.e.m.) compared to the control condition in two independent experiments. (TIF) [file pone.0073693.s001.tif]

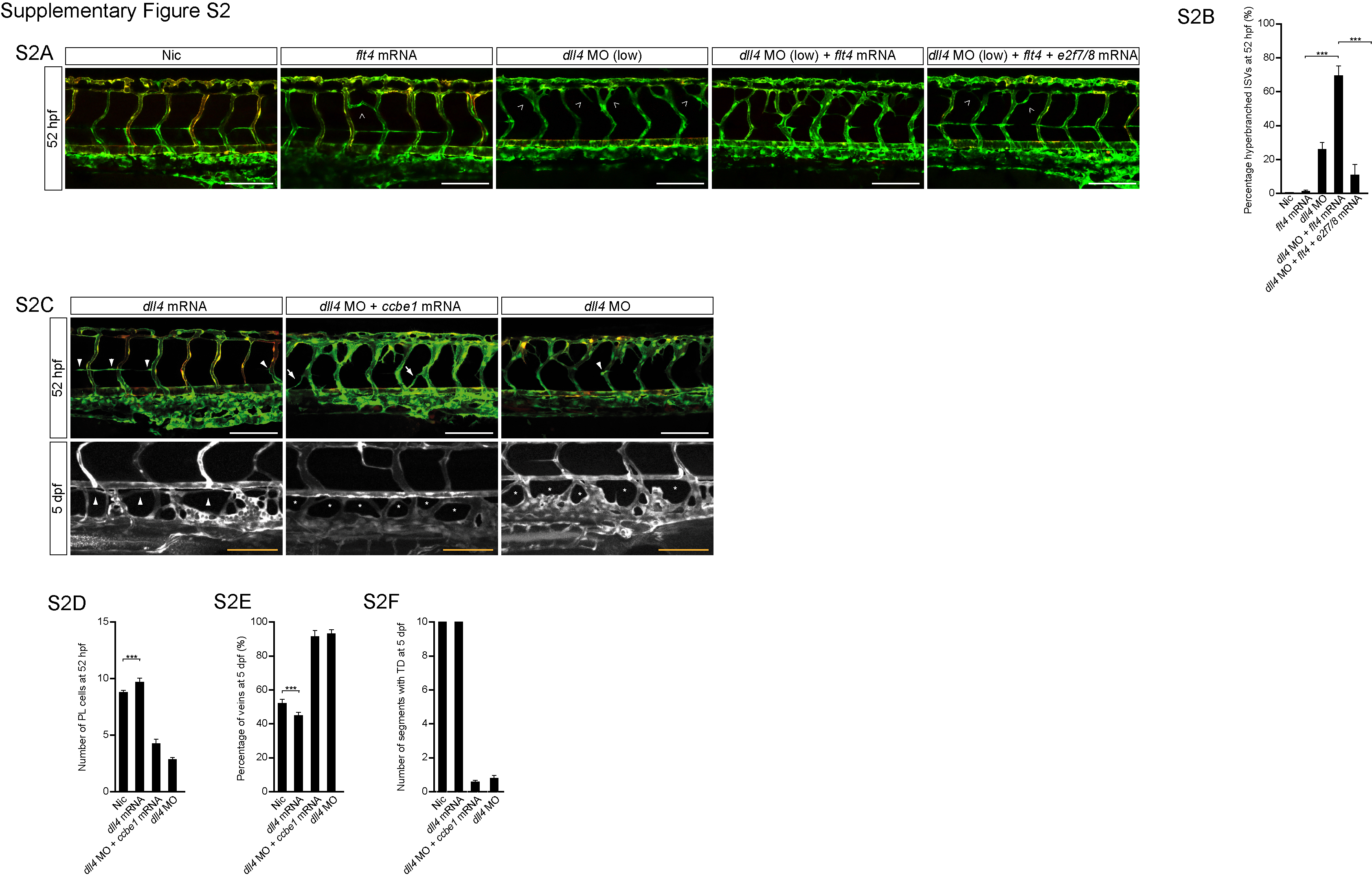

Supplement: Figure S2 — Hyperbranching and venous sprouting is dependent on proper Flt4 signaling. S2A Lateral images and quantification of Tg(fli1a:gfp;flt1enh:rfp) embryos treated as indicated and imaged at 52 hpf. S2C Lateral images and quantification of Tg(fli1a:gfp;flt1enh:rfp) embryos treated as indicated and imaged at 52 hpf. S2D, S2E, S2F, quantification of the indicated parameters. Concentrations: dll4 MO (low 1.5 ng in S2A and 3 ng in S2C–F); e2f7/8 mRNA (100 pg each); flt4 mRNA (100 pg); ccbe1 mRNA (100 pg). Arrows depict PLs that have connected to ISVs (S2C). Closed arrow heads indicate (upper panel in S2C) PLs or (lower panel, S2C) presence of the TD. Open arrowheads indicate hyperbranching ISVs. All scale bars are 100 µm. Stars indicate missing TD fragments. Data presented as the average (±s.e.m.) compared to the control condition in three independent experiments (*** P<0.001). At least n = 150 embryos per condition in three independent experiments were used for S2A–S2F. (TIF) [file pone.0073693.s002.tif]
